# Active case finding: comparison of the acceptability, feasibility and effectiveness of targeted versus blanket provider-initiated-testing and counseling of HIV among children and adolescents in Cameroon

**DOI:** 10.1186/s12887-018-1276-7

**Published:** 2018-09-25

**Authors:** Habakkuk Azinyui Yumo, Christopher Kuaban, Rogers Awoh Ajeh, Akindeh Mbuh Nji, Denis Nash, Anastos Kathryn, Marcus Beissner, Thomas Loescher

**Affiliations:** 1 R4D International Foundation, Yaounde, Cameroon; 2 Center for International Health (CIH), Ludwig-Maximilians-Universität, München, Germany; 3 Faculty of Health Sciences, University of Bamenda, Bamenda, Cameroon; 4 University of Yaounde I, Yaounde, Cameroon; 5 CUNY Graduate School of Public Health and Health Policy, New York, USA; 6 Department of Epidemiology & Population Health, Albert Einstein College of Medicine, New York, USA; 7 Montefiore Medical Center, New York, USA

**Keywords:** HIV, Identification, Children, Adolescents, Case detection, Linkage, Targeted PITC, Blanket PITC

## Abstract

**Background:**

Children and adolescents still lag behind adults in accessing antiretroviral therapy (ART), which is largely due to their limited access to HIV testing services. This study compares the acceptability, feasibility and effectiveness of targeted versus blanket provider-initiated testing and counseling (PITC) among children and adolescents in Cameroon.

**Methods:**

During a 6-month period in three hospitals in Cameroon, we invited HIV-positive parents to have their biological children (6 weeks-19 years) tested for HIV (targeted PITC). During that same period and in the same hospitals, we also systematically offered HIV testing to all children evaluated at the outpatient department (blanket PITC). Children of consenting parents were tested for HIV, and positive cases were enrolled on ART. We compared the acceptability, feasibility and effectiveness of targeted and blanket PITC using Chi-square test at 5% significant level.

**Results:**

We enrolled 1240 and 2459 eligible parents in the targeted PITC (tPITC) and blanket PITC (bPITC) group, and 99.7% and 98.8% of these parents accepted the offer to have their children tested for HIV, respectively. Out of the 1990 and 2729 children enrolled in the tPITC and bPITC group, 56.7% and 90.3% were tested for HIV (*p* < 0.0001), respectively. The HIV positivity rate was 3.5% (CI:2.4–4.5) and 1.6% (CI:1.1–2.1) in the tPITC and bPITC (*p* = 0.0008), respectively. This finding suggests that the case detection was two times higher in tPITC compared to bPITC, or alternatively, 29 and 63 children have to be tested to identify one HIV case with the implementation of tPITC and bPITC, respectively. The majority (84.8%) of HIV-positive children in the tPITC group were diagnosed earlier at WHO stage 1, and cases were mostly diagnosed at WHO stage 3 (39.1%) (*p* < 0.0001) in the bPITC group. Among the children who tested HIV-positive, 85.0% and 52.5% from the tPITC and bPITC group respectively, were enrolled on ART (*p* = 0.0018).

**Conclusions:**

The tPITC and bPITC strategies demonstrated notable high HIV testing acceptance. tPITC was superior to bPITC in terms of case detection, case detection earliness and linkage to care. These findings indicate that tPITC is effective in case detection and linkage of children and adolescents to ART.

**Trial registration:**

Trial registration Number: NCT03024762. Name of Registry: ClinicalTrial.gov. Date registration: January 19, 2017 (‘retrospectively registered’). Date of enrolment first patient: 15/07/2015.

**Electronic supplementary material:**

The online version of this article (10.1186/s12887-018-1276-7) contains supplementary material, which is available to authorized users.

## Background

Human immunodeficiency virus (HIV) case identification has been and remains a major obstacle to the expansion of antiretroviral therapy (ART) among infants, children and adolescents in sub-Saharan Africa due to multifaceted barriers at the patient, provider, community and national policy levels [1]. The uptake of early infant diagnosis (EID) using deoxyribonucleic acid-polymerase chain reaction (DNA-PCR) techniques for infants younger than 18 months of age is sub-optimal with a global coverage of 50% [2]. This gap is due to numerous barriers, including low antenatal consultation (ANC) attendance, weak supply chain management of pediatric HIV commodities, low retention, delayed test results, weak follow-up after delivery and poor linkage to treatment [3]. Implementation of the routine or blanket provider-initiated-testing and counseling (PITC), a strategy recommended by the World Health Organization (WHO) for HIV case finding among older children (≥18 months) is fragmentary. This situation is attributable to many factors, including fear of stigma, lack of staff training, lack of HIV testing kits, poor commitment from facility leadership, and missed parental consent to test children [4, 5].

As a result of these programmatic gaps, only approximately 10% and 15% of HIV-infected young (15–24 years) males and females, respectively, in Sub-Saharan Africa are aware of their HIV status [6]. As the gateway to HIV treatment and care, this low HIV testing uptake among children and adolescents translates to the current low pediatric ART coverage with only 43% of eligible children being on treatment compared to 54% of adults [7].

In Cameroon, the pediatric ART coverage gap is even wider, with only 18% of eligible children being on ART compared with 38% of adults [8]. This is happening despite the availability of HIV commodities (testing kits and antiretroviral drugs) provided free of charge for children by the government of Cameroon with the support of external funding agencies, most notably the Global Fund to fight HIV/AIDS, Tuberculosis and Malaria (GFATM) and the United States President’s Emergency Plan for AIDS Relief (PEPFAR). This gap indicates the need for alternative and/or innovative approaches to increase pediatric and adolescent HIV case identification and linkage to care in Cameroon and globally.

Given that over 90% [9] of pediatric HIV infections result from mother to child transmission, targeting with HIV testing, children of parents living with HIV/AIDS is a plausible high-yield case finding strategy as indicated by a study conducted in 2006 in Cameroon [10]. Though recommended by WHO since 2010 [11], implementation of this targeted PITC (tPITC) strategy is still sub-optimal in Cameroon and in other sub-Saharan African countries. Currently, there is a dearth of literature on the implementation outcome of tPITC, and most importantly, there is a lack of knowledge on its comparative advantage over the blanket PITC (bPITC). This study aimed to bridge this evidence gap and to contribute to the expansion of HIV treatment and care among children and adolescents.

## Methods

### Design

We conducted an interventional study in which we invited all parents living with HIV/AIDS receiving HIV care in three hospitals in Cameroon to have their children of unknown HIV status aged 6 weeks to 19 years to be tested for HIV (tPITC group). In the same hospitals, all parents/guardians who accompanied their sick children of the same age group for consultation at the outpatient departments were also counseled, and these children were invited to test for HIV irrespective of the presenting complaint (bPITC group).

### Setting

The study was conducted in the Limbe Regional Hospital (LRH), Ndop District Hospital (NDH) and Abong-Mbang District Hospital (ADH). These hospitals provide comprehensive health care services to the catchment population, including the management of HIV/AIDS. The study was conducted within the Active Search for Pediatric HIV/AIDS (ASPA) project, an initiative of Research for Development (R4D) International Foundation, a Cameroon-based global health research non-governmental organization. The ASPA project aimed to promote pediatric HIV service delivery through a range of activities, including capacity building of health personnel, services delivery both at facility and community level, nutritional support, monitoring and evaluation.

### Study period and population

Data were collected in the LRH from July to December 2015, and in ADH and NDH from June to November 2016. The study population in the tPITC group consisted of parents living with HIV/AIDS receiving care in the hospital and their children of unknown HIV status, aged 6 weeks to 19 years. Similarly, in the bPITC group, the study population consisted of parents/guardians and their sick children of the same age group who attended the hospital outpatient department for any reason. Children or parents critically ill (in vital distress) were excluded from the study.

### Study procedures

#### Site preparation

Prior to the study, input and support provided by the project to the respective hospitals included the following: staff training on both tPITC and bPITC activities, provision of HIV testing kits, and human resource support (dedicated staff to support project implementation).

#### Enrollment of participants and data collection

In the tPITC group, HIV-positive parents in care at the HIV treatment center (ART clinic) were counseled and invited by a trained counselor to participate in the study together with their children with unknown HIV status. These parents were offered a testing opportunity for their biological children in either the hospital or at home (community testing). In the bPITC group, parents/guardians were also counseled and invited to have their sick children tested for HIV irrespective of the reason of consultation.

**Fig. 1:**
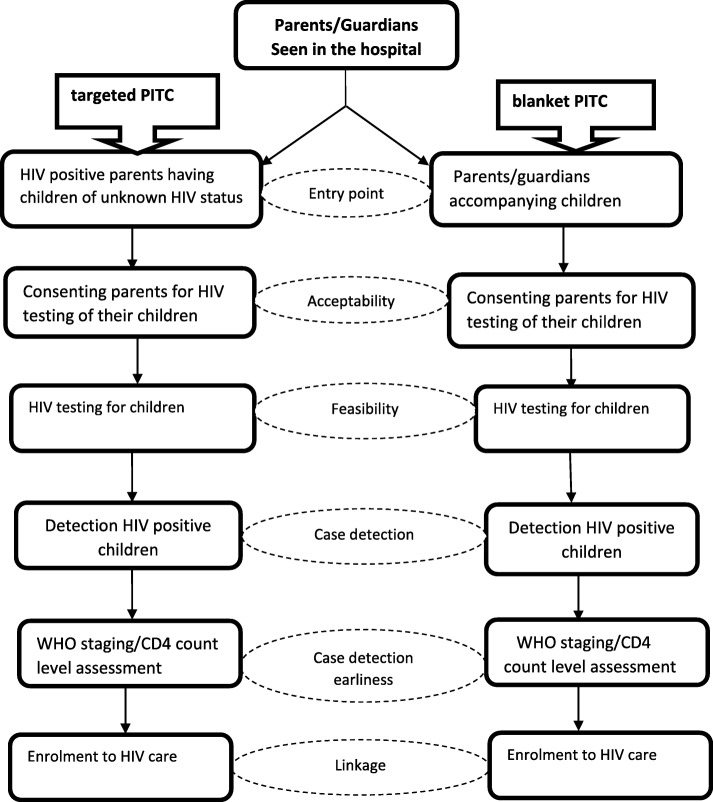
Enrollment, HIV testing and linkage to care and treatment of children and adolescents, ASPA Study, July–November 2016, Cameroon

In both groups, all parents/guardians who consented to participate in the study were enrolled together with their children. Pre-tested and structured questionnaires (Additional file 1: Questionaires 1–4) were used by a trained data clerk to collect socio-demographic information and the HIV/AIDS history of parents and children (Fig. 1). In the tPITC group, a sub-population of parents who initially agreed to bring their children for HIV testing, but subsequently did not, were interviewed using a structured questionnaire (Additional file 2: Questionaire 5) and this to determine the reason of their failure to bring children for testing.

#### HIV testing, linkage and ART enrolment

For children younger than 18 months of age, HIV testing was performed using DNA-PCR techniques. For children older than 18 months, HIV testing was performed using two HIV antibody rapid tests according to the Cameroon national guidelines. The WHO test and treat policy was not effective at the site level at the time of the study. Thus, children who tested positive for HIV were assessed for ART eligibility using WHO clinical staging and/or baseline biological analysis, including CD4 count. Eligible children were initiated on ART and monitored according to the Cameroon national guidelines.

### Sample size

We used the following formula to calculate the sample size for 2 proportions with dichotomous outcome [12]:

$$ \mathrm{N}={\left({\mathrm{Z}}_{\upalpha /2}+{\mathrm{Z}}_{\upbeta}\right)}^2\ast \left({\mathrm{p}}_1\left(1-{\mathrm{p}}_1\right)+{\mathrm{p}}_2\left(1-{\mathrm{p}}_2\right)\right)/{\left({\mathrm{p}}_1-{\mathrm{p}}_2\right)}^2, $$

Where: α = 5%, β = 20%, p_1_ = 10%, p_2_ = 5%. We found *N* = 432 children and adolescents per group and per hospital or 1296 per group for the three hospitals. Thus, a total of *n* = 1296 × 2 = 2592 children and adolescents for the two groups and three hospitals.

### Data management and analysis

Anonymous data from the questionnaires were entered into a database and analyzed using STATA 2013 (College Station, TX: StataCorp LP). The study outcomes were determined by computing the proportions and comparing the values using Chi-square test (X2) at 5% significant level.

### Definitions of terms

The study outcomes were defined and calculated as follows:

i) *Acceptability (acceptance rate):* proportion of parents who accepted to have their children tested among all eligible parents enrolled in the study
ii) *Feasibility (HIV testing uptake rate):* proportion of children who tested for HIV among all eligible children identified by the study
iii) *Effectiveness:* It was defined and measured as follows:
  - *HIV case detection/positivity rate:* proportion of HIV cases detected among children and adolescents tested for HIV
  - *HIV case detection earliness:* proportion of cases detected at WHO stage 1
  - *ART linkage rate*: proportion of cases linked to care or enrolled on ART

## Results

### Acceptability of tPITC and bPITC

The study offered enrolment to 3699 parents, including 1240 and 2459 in the tPITC and bPITC groups, respectively. In both groups, parents were predominantly from Ndop District Hospital (38.6%), followed by Limbe Regional Hospital (36.4%) and Abong-Mbang District Hospital (25.0%). Among these parents, 99.7% (1236/1240) and 98.8% (2430/2459) in the tPITC and bPITC, respectively, accepted to have their children tested for HIV.

### Feasibility of tPITC and bPITC

Through parents, 4719 eligible children were enrolled for HIV testing, including 1990 and 2729 in the tPITC and bPITC groups, respectively. In both groups, the children were predominantly from Ndop District Hospital Hospital (41.1%), followed by Limbe Regional Hospital (37.2%) and Abong-Mbang District Hospital (21.7%) (Table 1). None of the children enrolled had refused to be tested for HIV. Among the participating children, 56.7% (1129/1990) and 90.3% (2465/2729) (*p* < 0.0001) tested for HIV, respectively, in the tPITC and bPITC groups (Table 2). Among children ≤12 years, the HIV testing uptake (feasibility) rate was 60.5% compared to 91.2% (*p* < 0.0001), respectively, in the tPITC and bPITC groups. In comparison, among children older than 12 years of age, this rate was 43.8% vs 86.7% (*p* < 0.0001).

**Table 1:** Uptake of HIV services among children and adolescents in three hospitals in Cameroon, ASPA study, July 2015–November 2016

HIV services	tPITC				bPITC			
	Limbe	Abong-Mbang	Ndop	Total	Limbe	Abong-Mbang	Ndop	Total
	n (%)	n (%)	n (%)	n	n (%)	n (%)	n (%)	n
Children and adolescents enrolled	552 (27.7)	400 (20.1)	1038 (52.1)	1990	1205 (44.1)	623 (22.8)	901 (33.0)	2729
Children and adolescents tested for HIV in the hospital	257 (27.6)	212 (22.7)	462 (49.6)	931	951 (38.5)	619 (25.1)	895 (36.3)	2465
Children and adolescents tested for HIV in the community (only tPITC)	43 (21.7)	140 (70.7)	15 (7.5)	198	N/A	N/A	N/A	N/A
Children tested for HIV (both community and hospital)	300 (26.5)	352 (31.1)	477 (42.2)	1129	951 (38.5)	619 (25.1)	895 (36.3)	2465
Children and adolescents tested HIV+ in the community	0 (0.0)	1 (100)	0 (0.0)	1	N/A	N/A	N/A	N/A
Children and adolescents tested HIV+ in the hospital	5 (12.8)	13 (33.3)	21 (53.8)	39	14 (35.0)	21 (52.5)	5 (12.5)	40
Children and adolescents tested HIV+ (both hospital and community)	5 (12.5)	14 (35.0)	21 (52.5)	40	14 (35.0)	21 (52.5)	5 (12.5)	40
Children and adolescents initiated on ART	1 (2.9)	13 (38.2)	20 (58.8)	34	3 (14.2)	16 (76.1)	2 (9.5)	21

*N/A Not applicable*

**Table 2:** Acceptability and effectiveness of targeted versus blanket PITC in three hospitals in Cameroon, ASPA study, July 2015–November 2016

Outcome	tPITC				bPITC				P*
	Limbe	Abong-Mbang	Ndop	Total	Limbe	Abong-Mbang	Ndop	Total	
	% (n)	% (n)	% (n)	% (n)	% (n)	% (n)	% (n)	% (n)	
Acceptability rate	100.0 (327/327)	99.7 (344/345)	99.6 (566/568)	99.8 (1616/1619)	99.2 (1013/1021)	99.3 (575/579)	98.0 (842/859)	98.8 (2430/2459)	0.0005
Feasibility rate	54.3 (300/552)	88.0 (352/400)	46.0 (477/1038)	56.7 (1129/1990)	78.9 (951/1205)	99.4 (619/623)	99.3 (895/901)	90.3 (2465/2729)	< 0.0001
HIV positivity rate	1.7 (5/300)	4.0 (14/352)	4.4 (21/477)	3.5 (40/1129)	1.5 (14/951)	3.4 (21/619)	0.6 (5/895)	1.6 (40/2465)	0.0008
Linkage rate	20.0 (1/5)	92.9 (12/14)	95.2 (20/21)	85.0 (33/40)	21.4 (3/14)	76.2 (16/21)	40.0 (2/5)	52.5 (21/40)	0.0018

*p value comparing the outcome (total) of tPITC vs. bPITC in the 3 study sites

**Fig. 2:**
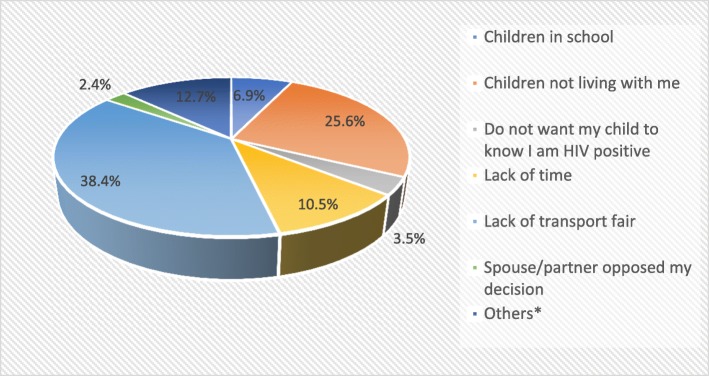
Reasons of PLHIV for not returning with children for HIV testing, ASPA study, July 2015–November 2016, Cameroon

The lack of transport fare (38.4%), children not living with biological parents (25.6%) and lack of time (10.5%) were the three primary reasons affecting the feasibility of tPITC strategy. These reasons were provided by a subgroup of 86 parents who initially accepted to have their children tested, but subsequently did not return to the hospital with their children for HIV testing (Fig. 2).

### HIV positivity/case detection

A total of 3594 children and adolescents were tested for HIV during the recruitment period; 1129 and 2465 in the tPITC and bPITC group, respectively (Table 1). The HIV positivity rate (case detection) was 3.5% (95% CI: 2.4–4.5) in tPITC group compared to 1.6% (95CI: 1.1–2.1) in the bPITC group (*p* = 0.0008) (Table 2). Among children ≤12 years, the HIV positivity rate was 3.3% vs 1.4% (*p* = 0.0006), respectively, in the tPITC and bPITC groups. In comparison, among children older than 12, this rate was 4.6% vs. 2.5% (*p* = 0.1621).

In the tPITC group, 17.of children were tested in the community and the hospital, respectively. The HIV positivity rate was 0.5% (1/198) in children tested in the community compared 4.2% (39/931) (*p* = 0.0107) among those tested in the hospital.

### Early detection of HIV cases

The proportion of HIV infected children diagnosed at WHO stage 1 and WHO stage 3 were 84.8% (28/33) and 15.2% (5/33) in the tPITC group, respectively, compared to 21.7% (5/23) and 39.1% (9/23) in the bPITC (*p* = 0.0001), respectively.

### Linkage to HIV care and treatment

In the tPITC group, 85.0% (34/40) of children tested HIV+ were linked to HIV treatment compared to 52.5% (21/40) of the cases in the bPITC (*p* = 0.0018) (Table 2). Among children ≤12 years, the linkage rate was 90.3% vs 58.6% (*p* = 0.005) in the tPITC and bPITC groups, respectively. Among children older than 12 years, this rate was 66.7% vs. 36.4% (*p* = 0.3698) in the tPITC and bPITC groups, respectively.

## Discussion

Applying the ambitious 90–90-90 target of the UNAIDS [13] to pediatrics would require global identification of 3.7 million infants, children and adolescents with HIV infection, treatment of 3.3 million, and achieving viral suppression among 3 million within the next four years [14]. Pediatric HIV case finding represents a major challenge in meeting these targets. The findings of this study add to the growing evidence that targeted strategies may increase HIV testing uptake, yield and linkage to treatment.

In the tPITC group, we found an HIV positivity rate (case detection rate/yield) of 3.5%, which was closer to the 4.0% but lower than the 7.4% reported by Saeed et al. in Malawi [15] and Wagner et al.in Kenya [16], respectively. The HIV prevalence (4.3%) in the general population in Cameroon (4.3%) [17] is lower compared to Malawi (9.2%) and Kenya (5.4%) [8] and this may explain the lower HIV positivity rate observed among the pediatric and adolescent population in our study compared to Malawi and Kenya as reported in the aforementioned studies. In the bPITC group, we found a prevalence of 1.6%, which was similar to the 1.8% reported by Zoufaly et al. in rural Cameroon [18] and closer to the 2.7% reported by Cohn et al. in a meta-analysis [19].

The HIV positivity rates reported by this study imply that the yield of newly identified HIV cases among children was two times higher with tPITC. To identify a new HIV case, 31 and 62 parents have to be counselled, and 29 and 63 children have to be tested, in the tPITC and bPITC groups, respectively. Therefore, less effort is needed with tPITC to identify a new pediatric or adolescent HIV case, and tPITC is more effective than bPITC in the context of our study.

The parents’ acceptance (acceptability) of HIV testing for their children was very high using both strategies (99.7% in tPITC vs 98.8% in bPITC). The slightly higher acceptance in the tPITC group may be due to enhanced HIV awareness resulting from the contact of these parents with HIV services. Ahmed et al. reported a similar high acceptability (93.5%) in their study in Malawi [15].

The uptake of HIV testing (feasibility) among children was significantly lower in the tPITC group (56.7% vs 90.3%, *p* < 0.0001). This may be attributable to the fact that the tPITC parents living with HIV were initially seen in the hospital in the first place for their own care, and their children were less likely to be present. Similarly, low uptake of HIV testing among children in tPITC was reported in Kenya where only 14% of parents who had initially consented to test children had followed through with the testing [16]. In our study, according to the parents’ declarations, the main reasons for their inability to return to the hospital with their children for HIV testing included the lack of transport fare (38.3%), children not living with them (25.6%), and the lack of time (10.5%). These reasons should be taken with caution because the HIV testing uptake could have also been limited by parental’ levels barriers, notably fear of self-disclosure, stigma and discrimination as reported by previous studies [4, 16, 20–22]. There is a need for qualitative research to provide in-depth information on parental barriers to the uptake of HIV testing for children in the context of tPITC approach implementation.

Although the HIV testing uptake was highest (90.3%) in the bPITC, nearly 10% of the children enrolled were not ultimately tested. This finding was attributable to a fraction of parents who initially consented to test their children, but they subsequently changed their decisions and did not go to the laboratory for testing. A number may have gone to the laboratory, but due to the long waiting time, they may have decided to leave without testing the child. Conducting the HIV testing on the spot or having a dedicated testing room for these children near the counseling office may have reduced the missed opportunity for testing.

In this study, pediatric HIV cases were diagnosed earlier in the tPITC group (84.8% at WHO stage 1) because this strategy tested asymptomatic children in contrast to the bPITC, in which children tested were evaluated for an illness (34.8% at WHO stage 2 and 39.1% at WHO stage 3). This finding was consistent with a previous targeted pediatric HIV testing in Malawi, where a large proportion (46.7%) of HIV infected children were diagnosed at WHO stage 1 [15]. Therefore, a pediatric HIV program could prioritize the tPITC strategy for early case identification as a means to reduce the high mortality rate associated with non-treatment of children living with HIV [23–25]. Linkage to care was significantly higher in the tPITC group (85.0% vs 52.5%, *p* = 0.0018). This finding may be explained by the fact that the large majority of parents were already in HIV care (96% of children were identified through parents on ART) and it was easier to link the children to HIV services because the parents, having seen the benefit of ART, quickly seized the treatment opportunity offered for their children who tested positive for HIV. This finding highlights the potential effect that prior enrolment of parents on ART could have on linkage of their children to care. This further demonstrates the effectiveness of the HIV care family-centered approach in enhancing pediatric HIV linkage and retention in care [26–29]. Nevertheless, in Limbe Regional Hospital, the linkage rate was statistically similar in the tPITC and bPITC groups (20.0% vs 21.4%, *p* = 1), but this rate was higher (but not statistically significant) in the tPITC group in Abong-Mbang (92.9% vs 76.2%, *p* = 0.2054) and significantly higher in Ndop (95.2% vs 40.0%, *p* = 0.0144) district hospital. In Abong-Mbang and Ndop district hospitals, we assigned staff members (linkage agents) to ensure that all children who tested positive for HIV were linked to care. Moreover, in these two new sites (through the humanitarian component of the ASPA project), nutritional kits were provided to HIV-positive children in care. Neither the linkage agent nor the nutritional support was provided at the Limbe Regional Hospital, which had the lowest linkage rate among the three sites. This finding suggests that both the linkage agent and nutritional support may have contributed meaningfully in improving linkage in the tPITC and bPITC groups. The positive effect of nutritional support in the linkage and retention of children in care has been previously demonstrated [30]. There is a need to further investigate this effect when combined with a linkage agent.

The limitations of this study were that the Limbe Regional Hospital began implementation in July 2015, while the Abong-Mbang and Ndop District Hospitals began later, in June 2016. We tweaked the implementation strategies in these 2 additional sites from lessons learned from the first site. In particular, we reinforced the follow-up of children diagnosed HIV+ to enhance linkage (introduction of a linkage agent). We also introduced the provision of nutritional kits to HIV+ children in care. These additional interventions may have contributed to increase the linkage rate in these 2 sites compared to Limbe. Thus, the results of Limbe Regional Hospital and that of Abong-Mbang and Ndop District Hospital are not comparable in all aspects. Nevertheless, because the primary objective of the study was not to compare the outcome per site, but rather, to compare the outcome of both tPITC and bPITC, the time difference in implementation per site did not affect the results of the study. In contrast, this stepwise implementation approach was found very useful because lessons learned from the first site (Limbe) informed the adjustments needed to have a more robust strategy for better linkage to care of HIV-positive children. Another potential limitation was that critically ill children were not included in our study. However, the number of these children coming to the hospital is usually marginal and their exclusion would not have affected our findings.

## Conclusions

The tPITC and bPITC strategies were highly acceptable to parents to support HIV testing for their children. The tPITC had a higher yield and provided an opportunity for early detection of pediatric and adolescent HIV cases as well as linkage to care before these children become sick and present to the health facility with HIV clinical manifestations.

However, the feasibility of tPITC strategy was lower compared to bPITC, which was due to the low HIV testing uptake among children and adolescents in the former strategy. The bPITC had a higher HIV testing uptake, but a lower linkage rate. Thus, the clinical cascade for the tPITC is challenged by the HIV testing uptake gap while that of the bPITC is constrained by the ART linkage gap.

Overall, the ASPA study demonstrated the superiority of tPITC over bPITC in terms of case detection, case detection earliness, and linkage to care and treatment. However, when the required resources are available, both strategies may be promoted to fast track the achievement of the ambitious 90–90-90 targets of the UNAIDS among children and adolescents by 2020. Meeting this objective would require the implementation of strategies that are suitable to optimize the outcome of both tPITC and bPITC approaches by improving the HIV testing uptake and linkage to care and treatment, respectively.

## Additional files

Additional file 1: Questionnaire No 1: Parents Living with Hiv/Aids. Questionnaire No 2: Parents/Guardians accompanying children to hospital. Questionnaire No 3: Enrolment form for children born to HIV positive parent(s). Questionnaire No 4: Enrolment form for children seen at the outpatients department. (DOCX 56 kb)

Additional file 2: Questionnaire No 5: Survey parents living with HIV/AIDS Questionnaire. (DOCX 20 kb)

## Abbreviations

ANC: Antenatal Consultations
ART: Antiretroviral therapy
ARV: Antiretroviral Drugs
ASPA: Active Search for Pediatric HIV/AIDS
bPITC: Blanket provider-initiated testing and counseling
CD4: Cluster of differentiation 4
CI: Confidence Interval
DBS: Dot blot spot
DNA: Deoxyribonucleic acid
EID: Early Infant Diagnosis
HIV/AIDS: Human Immunodeficiency Virus/Acquired Immune Deficiency Syndrome
LRH: Limbe Regional Hospital
MTCT: Mother to Child Transmission of HIV
NDH: Ndop District Hospital
OPD: Outpatient Department
PCR: Polymerase chain reaction
PITC: Provider-initiated testing and counseling
PLHIV: People Living with HIV/AIDS
PMTCT: Prevention of Mother to Child Transmission of HIV
R4D: Research for Development International
RT1: Rapid test 1
RT2: Rapid test 2
tPITC: Targeted provider- initiated testing and counseling
UNAIDS: The Joint United Nations Programme on HIV and AIDS
UNICEF: The United Nations Children’s Fund
WHO: The World Health Organization
