# Supplementary material for: Active case finding: comparison of the acceptability, feasibility and effectiveness of targeted versus blanket provider-initiated-testing and counseling of HIV among children and adolescents in Cameroon
Source: BMC Pediatr. 2018 Sep 25;18:309. doi: 10.1186/s12887-018-1276-7 (PMC6156944; doi:10.1186/s12887-018-1276-7)
Supplement: Supplementary file 1 — Questionnaire No 1: Parents Living with Hiv/Aids. Questionnaire No 2: Parents/Guardians accompanying children to hospital. Questionnaire No 3: Enrolment form for children born to HIV positive parent(s). Questionnaire No 4: Enrolment form for children seen at the outpatients department. (DOCX 56 kb) [file 12887_2018_1276_MOESM1_ESM.docx]

**QUESTIONNAIRE No 1 : PARENTS LIVING WITH HIV/AIDS**

*(Each parent/guardian should have only one form, irrespective of the number of children brought to the hospital)*

Study IDP| | | | | I I

Health Facility : ……………………………………………………..Date of encounter:…….../ …./…….

| 1. Full name | | | | | |  | | | |
| --- | --- | --- | --- | --- | --- | --- | --- | --- | --- |
| 2. Residence (village/quarter) | | | | | |  | | | |
| 3. Phone number | | | | | |  | | | |
| **The section above should be detached from this form prior to data entry** | | | | | | | | | |
| **ASPA QUESTIONNAIRE No 1: PLHIV Study IDP**\| \| \| \| \| I I I | | | | | | | | | |
| 4. Age | \| \| \| | | | 5. Sex | | ❑Male ❑Female | | | |
| 6. Education level | | ❑ None  ❑ nursery  ❑ primary  ❑ secondary  ❑ higher level | | | | 7. Occupation | | | ❑farming  ❑trading  ❑office work  ❑ student  ❑Others, specify:………………….. |
| 8. Marital status | | ❑Married  ❑Single  ❑Cohabitating  ❑Divorced/separated  ❑Widow/widower | | | | | | | |
| 9. Have you ever had TB | | ❑yes  ❑no | | | 10. Are you currently on  TB treatment? | | | ❑yes  ❑no | |
| 11. Are you on ARV  drugs? | | ❑yes  ❑no | | | 12.If yes, for how long  have you been on ARV  drugs | | | \| \| \| | |
| 13. How many children below 19 years do you have? | | \| \| \| | | | 14. Have you disclose your HIV status to your children? | | | ❑yes, to all of them  ❑yes, to some of them, specify why?..........................................................  ❑No, to none of them, specify why?.......................................................... | |
| 15. How many of your children less than 19 years have been tested for HIV?  If =0, go to no 19 | | | | | | | \| \| \| | | |
| 16. How many of them have tested HIV positive?  If =0, go to no 19 | | | | | | | \| \| \| | | |
| 17. How many of your HIV  positive children are receiving the following services? | | | CD4 count  monitoring | | | | \| \| \| | | |
|  |  |  | Antiretroviral drugs  (ARVs) | | | | \| \| \| | | |

|  | Cotrimoxazole  prophylaxis | \| \| \| | | |
| --- | --- | --- | --- | --- |
|  | nutritional support | \| \| \| | | |
|  | home visits by  community health workers | \| \| \| | | |
| 18. Are you | receiving nutritional  support from the treatment center? | ❑yes  ❑no | | |
|  | receiving home  visits by community health workers | ❑yes  ❑no | | |
|  | a member of an  association of people living with HIV/AIDS | ❑yes  ❑no | | |
|  | are you on antiretroviral drugs (ARV) | ❑yes  ❑no | | |
| 19. How many of your children  less than 19 years have not been tested for HIV? (this can be calculated = 13-15)  If = 0, end of questionnaire | ❑\| \| \|  ❑ don’t have children less than 19 years that have not been tested for HIV | | | |
| 20. Are you willing to have  these children tested for HIV? | ❑ No  ❑ Yes (go to 22 and then enrolment form for children) | | | |
| 21. Why don’t you want to  have them tested for HIV? |  | | | |
| 22. Where will you like to have your children tested? | ❑hospital  ❑community  ❑indifferent  ❑other, specify:………………………………… | | 23. How many children did this parent enrolled in  the study for HIV testing? (this question should be answered at the end of the enrolment period) | \| \| \| |

Comments:………………………………………………………………………………………………………………………………

Interviewed by:………………………………………….Signature……………………………….Date……………………………..

Checked by:……………………………………………..Signature………………………………..Date……………………………

**QUESTIONNAIRE No 2: PARENTS/GUARDIANS ACCOMPANYING CHILDREN TO HOSPITAL**

*(Each parent/guardian should have only one form, irrespective of*

*the number of children brought to the hospital)*

Study IDP| | | | | I I I I

Health Facility : …………………………………………... Date of encounter:…….../ …./…….

| 1.Full name | | | |  | | |
| --- | --- | --- | --- | --- | --- | --- |
| 2. Residence (village/quarter) | | | |  | | |
| 3. Phone number | | | |  | | |
| **The section above should be detached from this form prior to data entry** | | | | | | |
| **ASPA QUESTIONNAIRE NO 1: Parents at OPD Study IDP**\| \| \| \| \| I I I | | | | | | |
| 4. Age | \| \| \| | | 5. Sex | ❑ Male ❑ Female | | |
| 6. Education level | | ❑ None  ❑ nursery  ❑ primary  ❑ secondary  ❑ higher level | | 7. Occupation | | ❑farming  ❑trading  ❑office work  ❑ student  ❑Others, specify: ………………… |
| 8. Marital status | | ❑Married  ❑Single  ❑Cohabitating  ❑Divorced/separated  ❑Widow/widower | | | | |
| 9. What is your HIV status? | | ❑positive  ❑negative  ❑unknown | | | | |
| 10. How many children have you brought to the hospital today? | | | | | \| \| \| | |
| 11. Are you willing to have these children tested for  HIV today? | | | | | ❑yes , go to children enrollment form  ❑no if no, go to Q21 | |
| 12. Why don’t you want to have them tested for HIV  today? | | | | | ……………………………………………………… | |

Comments:………………………………………………………………………………………………………………………………...

Interviewed by:………………………………….Signature………..…………………………..Date…………………………………..

Checked by:……………………………………..Signature…………………………………...Date…………………………………...

**QUESTIONNAIRE No 3: ENROLLMENT FORM FOR CHILDREN BORN TO HIV POSITIVE PARENT(S)**

*(Each child should have a separate form)*

Health Facility:………………….………. Date of encounter:…………../..…./…………….

| Child’ full names: ……………………………………………………………………………………………  Study IDC\| \| \| \| \| I I I I | |
| --- | --- |
| **Parent’ details** | |
| Full names |  |
| Phone number |  |
| Residence |  |
| **The section above should be detached from this form prior to data entry** | |
| **ASPA QUESTIONNAIRE No 3: Children of PLHIV Study IDP\| \| \| \| \| I I I** | |
| ***A. Socio-demographic and HIV status of the child*** | |

| 1. Age | \| \| \| | | 2. Sex | ❑ Male ❑ Female | | | | | |
| --- | --- | --- | --- | --- | --- | --- | --- | --- | --- |
| 3. Identify for HIV  testing through | ❑Mother  ❑Father | | | | | | | | |
| 4. Education level | ❑ none  ❑ nursery  ❑ primary  ❑ secondary  ❑ higher level | | | | | | | | |
| 5. Did the mother attended antenatal consultations (ANC) during the pregnancy of this child? | ❑Yes  ❑No  ❑Unknown | | | | | | | | |
| 6. Where was the child born? | ❑Hospital  ❑ Home  ❑ Unknown | | | | 7. Was the mother received ARVs (PMTCT) drugs during pregnancy? | | | | ❑Yes  ❑ No  ❑ Unknown |
| 8. Has the child ever  tested for HIV? | ❑ yes  ❑ no (go to 9)  ❑ unknown (go to 9) | | | | 9. What is the HIV status of the child? | | | | ❑ Positive  ❑ Negative  ❑ unknown |
| 10. Is the child receiving  ARVs? | ❑Yes(go to Q10)  ❑No | | | | 11. Why is the child not in care? | | | |  |
| 12. Is the mother alive? | ❑ yes  ❑ no | 13. Mother’s education level | | | ❑ none  ❑ nursery  ❑ primary  ❑ secondary  ❑ higher level  ❑ Unknown | | | | |
| 14. Mother’s occupation | ❑ farming  ❑ trading  ❑ office work  ❑ others, specify:  ……..................... | 15. Mother's HIV status | | | ❑ negative  ❑ positive  ❑ unknown | | | | |
| 16. Is mother on ART? | ❑ yes  ❑ no  ❑ unknown | 17. Is the father alive? | | | ❑ yes  ❑ no | | | | |
| 18. Father’s education  level | ❑ None  ❑ nursery  ❑ primary  ❑ secondary  ❑ higher level  ❑ unknown | 19. Father’s occupation | | | ❑ farming  ❑ trading  ❑ office work  ❑ Others,specify: ………………… | | | | |
| 20. Father’s HIV  status | ❑ Negative  ❑ Positive  ❑ unknown | 21. Is father on  ART? | | | ❑ yes  ❑ no  ❑ unknown | | | | |
| 22. Mode of recruitment | Parents from:  ❑ ARVs  ❑ VCT  ❑ PTMCT  ❑ TB unit  ❑ LTF  Others….. | 23. Did the mother take  ARVs drugs during  the pregnancy of this child? | | | | ❑ yes  ❑ no  ❑ unknown | | | |
| 24. Preferred site for HIV testing? | ❑ Hospital  ❑ Community  ❑ Indifferent | 25. Actual site for HIV  testing? | | | | | ❑ Hospital  ❑ community | | |
| 26. 1st rapid HIV  test result | ❑ Negative  ❑ Positive  ❑Indeterminate | 27. 2nd rapid HIV test  result | | | | | ❑Negative  ❑Positive  ❑Indeterminate | | |
| 28. PCR testing  (if child <18 months) | ❑Negative  ❑Positive❑ | 29. Final HIV results | | | | | | ❑Negative  ❑Positive  ❑Indeterminate | |

**B. ART ELIGIBILITY ASSESSMENT AND LINKAGE TO CARE (*only for HIV+ children*)**

| 30. Clinical assessment | Weight: . (kg) Height: . (cm)  Head Circumference: . (cm) WHO Staging:  ❑ Stage 1 ❑ Stage 2  ❑ Stage 3 ❑ Stage 4 | | 31.Laboratory  Assessment | | Hb (g/dl): . TLC: CD4:  %CD4: |
| --- | --- | --- | --- | --- | --- |
| 32. Immunological Classification: | | ❑ No evidence of suppression (%CD4≥25)  ❑ Evidence of moderate suppression (15≤%CD4≤24)  ❑ Severe suppression (%CD4<15) | | | |
| 33. Eligible to ART | ❑ No*(go to Q31)*  ❑ Yes | 34. ART regimen prescribed | | ❑ NVP-based  ❑ EFV-based  ❑ PI-based | |
| 35. Registration in pre-ART register | ❑ Yes  ❑ No | 36. specify the  reasons for the non registration in pre-ART register | | ❑ Lost to follow up  ❑ No register  ❑ Others, specify: ………...... | |
| 37. Cotrimoxazole prescribed | ❑ yes  ❑ no, specify reasons: ……….......................................................................................... | | | | |

Comments:………………………………………………………………………………………………………………………………...

Interviewed by:………………………………….Signature………..…………………………..Date…………………………………..

Checked by:……………………………………..Signature…………………………………...Date…………………………………...

**QUESTIONNAIRE No 4: ENROLMENT FORM FOR CHILDREN SEEN AT THE OUTPATIENT DEPARTMENT**

*(Each child should have a separate form)*

| Child’ full names: ………………………………………………………………………………………....  Study IDC\| \| \| \| \| I I I I | |
| --- | --- |
| Parent’ details | |
| Full names |  |
| Phone number |  |
| Residence |  |
| The section above should be detached from this form prior to data entry | |
| ASPA QUESTIONNAIRE No 4: Children of PLHIV Study IDP\| \| \| \| \| I I I | |

| 1. Age | | \| \| \| | | 2. Sex | | | ❑ Male ❑ Female | | |
| --- | --- | --- | --- | --- | --- | --- | --- | --- | --- |
| 3. Brought to the  hospital by | | ❑Mother  ❑Father  ❑Grand-mother  ❑Grand-father  ❑Others, specify: ………................................................ | | | | | | | |
| 4. Education level | | ❑ none  ❑ nursery  ❑ primary  ❑ secondary  ❑ higher level | | | | | | | |
| 5. Did the mother attended antenatal consultations (ANC) during the pregnancy of this child? | | ❑Yes  ❑No  ❑Unknown | | | | 6. Where was the child born? | | | ❑ Hospital  ❑ Home  ❑ Unknown |
| 7. Has the child ever tested for HIV? | | ❑ yes  ❑ no (go to no 7)  ❑ unknown (go to no 7) | | | | 8. What is the HIV  status of the child? | | | ❑ negative (go to  Q9)  ❑ positive  ❑ unknown |
| 9. If positive, is the  child on ARVs | | ❑ Yes ( go to Q9)  ❑ No | | | | 10. Why is the  child not on  ART | | |  |
| 11. Is the mother alive? | | | ❑ yes  ❑ no | | 12. Mother’s  education level | | | ❑ none  ❑ nursery  ❑ primary  ❑ secondary  ❑ higher level  ❑ Unknown | |
| 1. 13. Mother’s occupation | | | ❑ farming  ❑ trading  ❑ office work  ❑ Others, specify:…… | | 14. Is the  father alive? | | | ❑ yes  ❑ no | |
| 15. Father’s education level | | | ❑ None  ❑ nursery  ❑ primary  ❑ secondary  ❑ higher level  ❑ unknown | | 16. Father’s occupation | | | ❑ farming  ❑ trading  ❑ office work  ❑ Others, specify:………………… | |
| 17. 1st rapid HIV test result | | | ❑ Negative  ❑ Positive  ❑ Indeterminate | | 18. 2nd rapid HIV test result | | | ❑Negative  ❑Positive  ❑Indeterminate | |
| 19. PCR (if child  <18 months) | ❑Negative  ❑Positive | | 20. Final HIV status | | | | | ❑ Negative (stop here)  ❑ Positive  ❑ Indeterminate | |

**B. ART ELIGIBILITY ASSESSMENT AND LINKAGE TO CARE (*only for HIV+ children*)**

| 21. Clinical Assessment | Weight: . (kg)  Height: . (cm)  Head Circumference: . (cm) WHO Staging:  ❑ Stage 1 ❑ Stage 2  ❑ Stage 3 ❑ Stage 4 | | 22.Laboratory  Assessment | | Hb (g/dl): .  TLC: CD4:  %CD4: |
| --- | --- | --- | --- | --- | --- |
| 23. Immunological Classification: | | ❑ No evidence of suppression (%CD4≥25)  ❑ Evidence of moderate suppression (15≤%CD4≤25)  ❑ Severe suppression (%CD4<15) | | | |
| 24. Eligible to ART | ❑ No*(go to Q26)*  ❑ Yes | 25. ART regimen  prescribed | | ❑ NVP-based  ❑ EFV-based  ❑ PI-based | |
| 26. Registration in  pre-ART  register | ❑ Yes  ❑ No | 27. specify the  reasons for the non registration in pre-ART register | | ❑ Lost to follow up  ❑ No register  ❑ Others, specify: ………...... | |
| 28. Cotrimoxazole  prescribed | ❑ yes  ❑ no, specify reasons: ………............................................. | | | | |

Comments:………………………………………………………………………………………………………………………………...

Interviewed by:………………………………….Signature………..…………………………..Date…………………………………..

Checked by:……………………………………..Signature…………………………………...Date…………………………………...
