# Supplementary material for: Active case finding: comparison of the acceptability, feasibility and effectiveness of targeted versus blanket provider-initiated-testing and counseling of HIV among children and adolescents in Cameroon
Source: BMC Pediatr. 2018 Sep 25;18:309. doi: 10.1186/s12887-018-1276-7 (PMC6156944; doi:10.1186/s12887-018-1276-7)
Supplement: Supplementary file 2 — Questionnaire No 5: Survey parents living with HIV/AIDS Questionnaire. (DOCX 20 kb) [file 12887_2018_1276_MOESM2_ESM.docx]

**QUESTIONNAIRE No 5.**

**SURVEY PARENTS LIVING WITH HIV/AIDS**

| 1. What is the travel time in a vehicle from your home to the hospital? | | ❑<30 min ❑30min-60min ❑>60min |
| --- | --- | --- |
| 1. What are the reasons for not bringing your child to the hospital for HIV testing? | | ❑ Lack of transport money  ❑ Lack of time  ❑ Children in school  ❑ Children not living with me  ❑ Spouse/partner opposed my decision  ❑ Do not want my child to know I am HIV positive  ❑ Child too small  ❑ Others:…………………............................. |
| 1. If applicable, what can be done for you to bring your child in the hospital for HIV testing? | | |
| 1. Will you be willing for community health workers to come and test your child at home? | ❑Yes  ❑No  ❑ Undecided  ❑ I have to ask my spouse/partner | |
| 1. Have you disclosed your status to your spouse/partner | ❑Yes  ❑No  ❑Do not remember  ❑Choose not to anwser | |

Study IDP|___|___|___|___|
